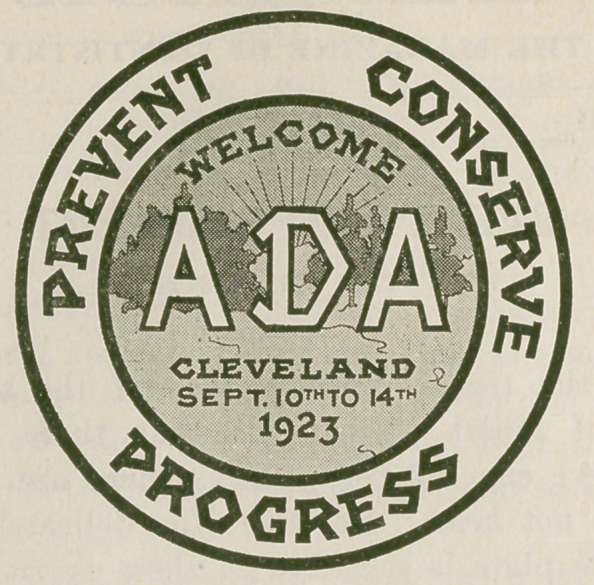# American Dental Association

**Published:** 1923-05

**Authors:** 


### AMERICAN DENTAL ASSOCIATION

The above seal of the meeting of the American Dental Association in Cleveland was designated by the Local Committee of Arrangements especially for that event.

Its outstanding feature is the wonderful slogan.

*Prevent* *Conserve* *Progress*

Upon each of these words the Dental Professiori today are standing in bold relief.

Progress in human health is to prevent disease and conserving vitality is the opposite of destruction. Therefore the slogan is one of great importance to this meeting. It will be the key note.

The inner portion of the seal bears the word ''Welcome'' which is significant of the hospitality of the dental profession of Cleveland.

Cleveland bears a wonderful reputation for a convention city, with its new Auditorium which will be an inovation to many for its facilities, and the many good hotels, accessibility by rail and water will bring the largest attendance to any meeting ever held.

Reduced railroad rates and no advanced hotel rates are certain.

Now is the time to arrange your vacation which will include the American Dental Association.

## Figures and Tables

**Figure f1:**